# Reinterpretation of Conflicting ClinVar *BRCA1* Missense Variants Using VarSome and CanVIG-UK Gene-Specific Guidance

**DOI:** 10.3390/diagnostics14242821

**Published:** 2024-12-14

**Authors:** Min-Kyung So, Gaeul Jung, Hyun-Jeong Koh, Sholhui Park, Tae-Dong Jeong, Jungwon Huh

**Affiliations:** 1 Department of Laboratory Medicine, Ewha Womans University College of Medicine, Seoul 03760, Republic of Korea; mkso@ewha.ac.kr (M.-K.S.); solheepark@ewha.ac.kr (S.P.); tdjeong@ewha.ac.kr (T.-D.J.); 2 Department of Genetic Counseling, Ewha Womans University College of Medicine Graduate School, Seoul 03760, Republic of Korea; mystum@naver.com (G.J.); kwsa.hjkoh@gmail.com (H.-J.K.)

**Keywords:** *BRCA1* missense variants, conflicting interpretation, ClinVar, *BRCA1/BRCA2*: CanVIG-UK gene-specific guidance, VarSome

## Abstract

Background: The accurate interpretation of the *BRCA1*/2 variant is critical for diagnosing and treating hereditary breast and ovarian cancers. ClinVar is a widely used public database for genetic variants. Conflicting classifications of pathogenicity can occur when different submitters categorize the same genetic variant inconsistently as pathogenic (PV), likely pathogenic (LPV), likely benign (LBV), benign (BV), or a variant of uncertain significance (VUS). The conflicting ClinVar *BRCA1/2* variant classifications hinder clinical decision making. We reinterpreted 450 *BRCA1* missense variants with conflicting interpretations in ClinVar (accessed on 20 December 2022). Methods: VarSome and the BRCA1/BRCA2: CanVIG-UK gene-specific guidance (CanVIG-UK) classifications were compared, and the five original classifications were consolidated into three categories (PV/LPV, VUS, and BV/LBV). Consensus analysis was performed between re-extracted ClinVar data and VarSome and CanVIG-UK results. Results: The three-category classification of the variants resulted in an overall concordance rate of 58.9% for *BRCA1* missense variant interpretation between CanVIG-UK and VarSome, with VarSome having rates of 11.3, 24.7, and 64.0% for PV/LPV, VUS, and BV/LBV classifications and CanVIG-UK having rates of 11.1, 51.6, and 37.3% for P/LPV, VUS, and BV/LBV classifications, respectively. No variants classified as PV/LPV in VarSome were classified as BV/LBV in CanVIG-UK and vice versa. By 1 May 2024, 3.8% (17/450) of these conflicting variants reached a consensus classification in ClinVar and were definitively classified (9 PV/LPV, 1 VUS, and 7 BV/LBV). Conclusions: VarSome and CanVIG-UK have different features that help improve the accuracy of pathogenicity classification, highlighting the potential complementary use of both tools to support clinical decision making.

## 1. Introduction

*BRCA1* and *BRCA2* genes play a critical role in DNA repair, and individuals who inherit pathogenic variants (PVs) in these genes face a substantially increased risk of developing breast and ovarian cancers [1,2]. For patients with metastatic or recurrent cancers carrying these variants, PARP inhibitors like Olaparib present a treatment option. High-risk individuals may also consider preventive measures, such as mastectomy or oophorectomy, and regular screenings for breast and ovarian cancers. Additionally, genetic testing and counseling for family members to identify *BRCA1* and *BRCA2* PVs are important for risk management. The accurate identification of these variants is therefore essential, and genetic testing has become an important part of clinical practice.

ClinVar (https://www.ncbi.nlm.nih.gov/clinvar/) is a widely used public database for genetic variants, providing accessible information on genetic variations for researchers and clinicians. As of November 2024, ClinVar hosts approximately 4.7 million records with classifications submitted by around 2940 contributors, and this dataset continues to grow. Despite ClinVar’s data quality control measures, submissions from multiple submitters sometimes result in conflicting interpretations, leading to inconsistent categorization as a PV, likely pathogenic variant (LPV), likely benign variant (LBV), benign variant (BV), or variant of uncertain significance (VUS).

Among various types of *BRCA1* and *BRCA2* variants, missense variants are particularly challenging to interpret due to factors such as experimental functional data, variant location, and computational prediction models [3,4]. Consequently, different submitters may classify the same missense variant differently in ClinVar, resulting in conflicting interpretations [5]. These inconsistencies present a challenge for clinicians in planning effective treatment and prevention strategies. If conflicting classifications result in a variant being incorrectly classified as PV/LPV, unnecessary clinical interventions may occur, potentially leading to unwarranted preventive surgeries or excessive treatments. Conversely, variants misclassified as BV/LBV or VUS may delay appropriate surveillance or treatment, potentially missing critical opportunities for preventive measures in high-risk individuals. Such discrepancies not only increase uncertainty but also induce anxiety and confusion, complicating the decision-making process for both clinicians and patients.

To address these challenges, various guidelines and tools have been developed to assist variant interpretation [6,7]. Among these, the most widely recognized are the American College of Medical Genetics and Genomics (ACMG) guidelines, which provide general frameworks for variant classification [7]. The ACMG guidelines play a crucial role in standardizing variant interpretation; however, the explicit details regarding the weighting and combination of individual evidence items remain ambiguous, often requiring expert judgment. Consequently, variant interpretation frequently relies on the experience and discretion of individual experts. To address these limitations, clear and objective evidence application guidelines, as along with automated tools, are being introduced. VarSome is a powerful and widely used tool that integrates data from many sources, including population databases and functional prediction algorithms, thus providing a comprehensive and automated interpretation of genetic variants across multiple genes. Its extensive data integration and automated analysis make it an invaluable resource for initial variant classification [8,9]. However, the complexity of *BRCA1* variants has led to the development of more specialized gene-specific guidance, such as the *BRCA1/BRCA2*: CanVIG-UK gene-specific guidance (CanVIG-UK) [10]. CanVIG-UK is specifically tailored to interpret *BRCA1* and *BRCA2* variants, incorporating gene-specific evidence that broader guidelines may not fully address.

This study focused on the reinterpretation of 450 *BRCA1* missense variants with conflicting interpretations in ClinVar (accessed on 20 December 2022) using two tools for variant interpretation: VarSome and CanVIG-UK. This study aimed to evaluate their respective strengths and limitations through systematic comparison, ultimately offering critical insights for enhancing clinical decision making.

## 2. Materials and Methods

### 2.1. ClinVar Data Extraction

**Figure 1:**
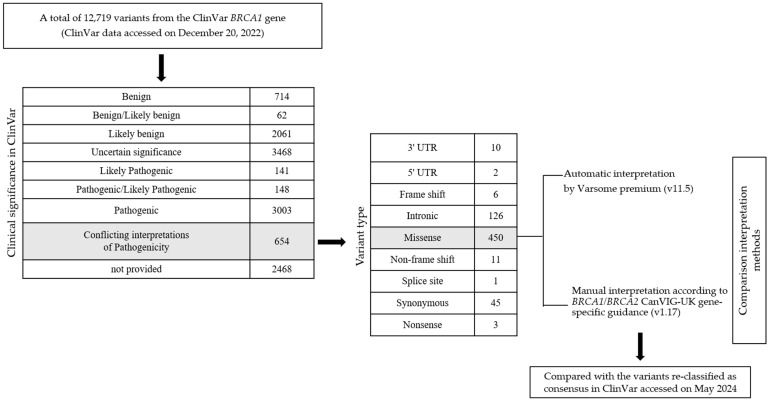
Workflow for interpretation and comparison of conflicting ClinVar *BRCA1* missense variants between VarSome and CanVIG-UK.

A total of 12,719 variants of the *BRCA1* gene were extracted from the ClinVar database (accessed on 20 December 2022). Of these, 654 variants were identified to have conflicting interpretations of pathogenicity. From this subset, we selected missense variants, resulting in 450 missense variants for further analysis (Figure 1). We also analyzed the number of submitters to the ClinVar database who provided interpretations for each variant, counting only those in which the assertion criteria were specified in the review. We re-extracted the same 450 conflicting missense variants from ClinVar on 1 May 2024, to verify whether any of them had achieved consensus classifications, i.e., being definitively categorized into one of the following groups: PV/LPV, VUS, or BV/LBV.

### 2.2. Variant Classification

#### 2.2.1. CanVIG-UK

Each *BRCA1* missense variant was manually classified according to the *BRCA1/BRCA2*: CanVIG-UK gene-specific guidance (v1.17) by a genetic specialist at our institution [10]. Subsequently, a peer genetic specialist from the same institution performed a cross-validation to review and verify the variant interpretations. The guidance is specifically designed for *BRCA1* and *BRCA2* variants, incorporating gene-specific considerations that enhance classification accuracy. Additionally, the classification process was based on the CanVIG-UK Consensus Specification for Cancer Susceptibility Genes (v2.17), published by the CanVIG-UK Working Group, which ensures adherence to consensus-driven standards in variant interpretation [11]. We classified missense variants by applying a combination of the evidence criteria, including PS1, PS3 (strong to supporting), PS4 (very strong to supporting), PM1 (moderate to supporting), PM2 (moderate to supporting), PM5 (moderate to supporting), PP3, PP5 (very strong to supporting), BA1, BS1, BS3, BP1, and BP4, applying this standard while disregarding PM2 evidence when benignity was strongly supported.

We briefly explain the main evidence codes applied in CanVIG-UK as follows:

- For population database evidence, we applied PM2, BA1, and BS1 criteria, using allele frequency thresholds and cancer-free female controls of all ethnicities from the Genome Aggregation Database (gnomAD), while acknowledging the low penetrance of PVs in male carriers. For PM2, we based the analysis on the absence (PM2) or extremely low frequency (PM2_sup) in large control databases, specifically focusing on cancer-free female controls. For BA1 and BS1, the maximum tolerated allele frequency thresholds were set at 0.001 (0.1%) and 0.0001 (0.01%), respectively, filtering allele counts calculated using the upper 95% confidence interval. Calculations utilized resources such as CardioDB https://www.cardiodb.org/allelefrequencyapp/ (accessed on December 2022).
- For functional evidence, we applied the PS3 and BS3 criteria based on well-established in vitro and in vivo functional studies, in alignment with the CanVIG functional assay scores in the CanVIG-UK guidance [12,13,14,15]. When a missense variant was reported to affect splicing in a functional study, the PS3 (splice effect) evidence code was applied.
- For amino acid changes at the same position, we applied PM5 criteria for missense variants, where a different missense change at the same amino acid residue had previously been classified as pathogenic. PM5 was used at a moderate level if the variant under examination had an equivalent or more deleterious REVEL score compared to the reference variant. PM5 was applied at a supporting level if the reference variant was classified as LPV and had limited reports, or if the variant had a less deleterious REVEL score than the reference variant.
- For disease-related approaches, we applied PP5 and BP6 criteria utilizing published multifactorial analysis data, including likelihood ratios and log likelihood ratios, as comprehensive data sources to represent cumulative evidence [16,17].
- For protein in silico function assessment, we applied PP3 and BP4 criteria for protein impact evaluation using the meta-predictor REVEL. For PP3, variants with a REVEL score greater than 0.7 were classified as having a potentially damaging impact on the protein. Conversely, for BP4, variants with a REVEL score below 0.4 were considered to have a low impact, supporting a benign classification.
- According to the variant position, we applied PM1 and BP1 criteria to assess the pathogenicity of missense variants in *BRCA1*. For PM1, we used supporting (PM1_sup) or moderate (PM1_mod) evidence levels for variants located in specific functional domains, including the *BRCA1* RING (amino acids 1–101), BRCT (amino acids 1650–1863), and COILED-COIL (amino acids 1391–1424). Specific residues within these domains were assessed with PM1_mod, where missense changes are known to have a significant impact. For BP1, we applied supporting evidence for missense variants outside these key domains if no splicing effects (<0.2) were predicted.
- For case–control evidence, we applied PS4 criteria to assess the prevalence of BRCA1 variants in affected individuals compared to control populations. We used an odds ratio threshold of OR ≥ 10 to identify enriched case series. It should be noted that due to limited literature searches, some variants may not have been thoroughly reviewed across all available case–control data sources.

Points were calculated for each criterion based on the strength of evidence—supporting (1 point), moderate (2 points), strong (4 points), and very strong (8 points)—resulting in a cumulative score for variant classification. This system ensures consistent classification of variants into one of the following categories: PV (≧10), LPV (6~9), VUS (0~5), LBV (−1~−5), and BV (≦−6). Following recommendations, PM2 (PM2_sup) was excluded from the calculation of the net exponent total for benignity when no additional pathogenicity-related evidence was present. If the net exponent score exceeded the benignity threshold and included two or more pathogenicity-related evidence elements, the variant was categorized as VUS. The methodology of this scoring system is detailed in the CanVIG-UK consensus recommendations [18].

#### 2.2.2. VarSome

The tier classification of each variant was determined using VarSome premium (v11.5), an automated variant interpretation tool that applies the ACMG guidelines [8]. VarSome integrates data from multiple sources, including population databases, such as gnomAD with specific frequency thresholds and functional prediction algorithms, such as MetaRNN for missense variants (additional information available at https://updates.VarSome.com/en/VarSome-11.5). Pathogenicity was assessed by assigning points to each rule based on evidence strength—supporting (1 point), moderate (2 points), strong (4 points), and very strong (8 points)—and calculating a total score to determine the variant classification. This tool automatically synthesizes the information to classify variants into five categories—PV (≧10), LPV (6~9), VUS (0~5), LBV (−6~−1), and BV (≦−7)—using the point system [19]. In this study, only the automatically classified results provided by VarSome were analyzed, and no manual adjustments were made to the classification.

### 2.3. Comparison of Classification Results

The classification results obtained from both CanVIG-UK and VarSome were compared to identify discrepancies and overall concordance rates using MedCalc^®^ Statistical Software version 20.211. To facilitate a more coherent comparison, the five classifications were consolidated into three broad categories: PV/LPV, VUS, and BV/LBV. For consensus analysis, we compared the re-extracted ClinVar data (accessed on 1 May 2024) with the results obtained from VarSome and CanVIG-UK to assess their concordance.

## 3. Results

### 3.1. Conflicting BRCA1 Variants in ClinVar

We analyzed 450 missense variants from the ClinVar database with conflicting interpretations of pathogenicity as of 20 December 2022. These variants were predominantly located within the *BRCA1* RING domain (amino acids 2–101) encoded by exons 2–5 and the BRCT domain (amino acids 1650–1857) encoded by exons 15–23 (Figure S1). In ClinVar, a total of 450 variants with conflicting interpretations were submitted by between two and 15 submitters per variant. The most common scenario involved 91 variants reported by two submitters, followed by 85 variants reported by three submitters, and 73 variants reported by five submitters (Table S1).

### 3.2. Variant Calssification Using Varsome and CanVIG-UK

The classification of the variants into three categories (PV/LPV, VUS, and BV/LBV) resulted in VarSome having rates of 11.3% for PV/LPV, 24.7% for VUS, and 64.0% for BV/LBV and CanVIG-UK having rates of 11.1% for PV/LPV, 51.6% for VUS, and 37.3% for BV/LBV classifications (Table 1 and Table S1). In VarSome, 64.0% of cases were classified as BV/LBV, while in CanVIG-UK, the largest portion, 51.6%, was classified as VUS.

### 3.3. Comparison of Classification Results Between Varsome and CanVIG-UK

**Figure 2:**
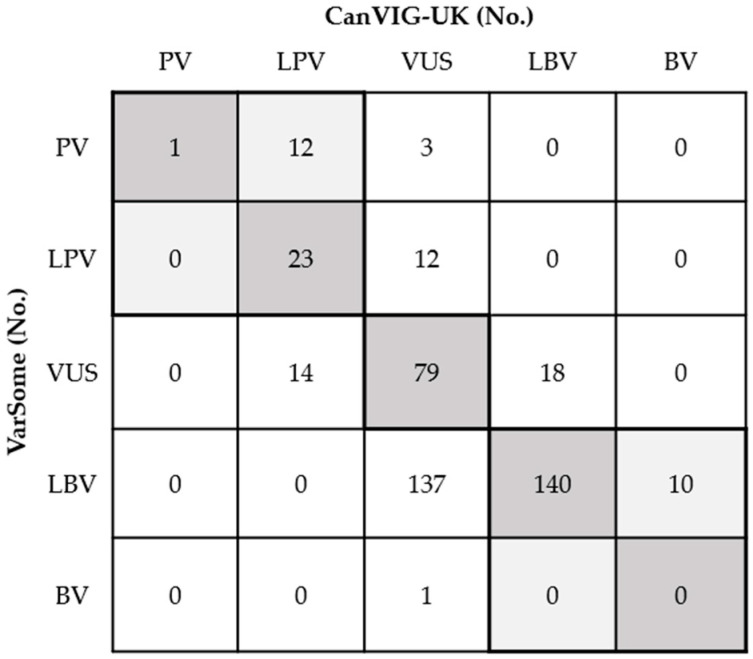
Comparison of classification of 450 conflicting variants in ClinVar (accessed on 20 December 2022) between VarSome and CanVIG-UK. Variants within the bolded lines were considered concordant (overall concordance rate of 58.9%; 95% CI: 52.0–66.4%).

The overall concordance rate was 58.9% (95% CI: 52.0–66.4%; 265/450) between VarSome and CanVIG-UK (Figure 2). No variants classified as PV/LPV in VarSome were classified as BV/LBV in CanVIG-UK or vice versa. Discrepancies were particularly evident in the classification between VUS and LBV, wherein 137 variants were classified as LBV by VarSome but as VUS by CanVIG-UK, whereas 18 variants showed the opposite trend (Figure 2). Additionally, CanVIG-UK classified 15 variants as VUS, whereas VarSome classified these at a high level as PV in 3 variants and LPV in 12 variants. Eleven of these variants were assigned to the BS3 functional benign evidence code in CanVIG-UK. For the remaining four variants, VarSome applied additional evidence by either using or strengthening the PM5 code to PM5_strong, suggesting a pathogenic association that was not recognized at the same level in CanVIG-UK (detailed data in Table S2). Meanwhile, in CanVIG-UK, 14 variants were classified as LPV, while VarSome classified them as VUS, showing a discrepancy in classification. This discrepancy was mainly due to CanVIG-UK applying functional pathogenic evidence codes (PS3). Each of these variants had also been reported as PV/LPV by at least one submitter in ClinVar (detailed data in Table S3).

### 3.4. Proportion of VarSome and CanVIG-UK Classifications by ClinVar Submissions

We evaluated the proportions of VarSome and CanVIG-UK classifications in relation to the ClinVar submitter results (Table 2). When more than 50% of the ClinVar submitters classified a variant as PV/LPV, VarSome and CanVIG-UK classified 90.5% and 95.2% of the variants as PV/LPV, respectively. Conversely, when 50% or fewer of the ClinVar submitters classified a variant as PV/LPV, 88.2% were classified the same by CanVIG-UK, whereas only 52.9% were classified the same by VarSome. For variants classified as PV/LPV by at least one ClinVar submitter, CanVIG-UK classified 90.9% (50/55) as PV/LPV and the remaining 9.1% (5/55) as VUS. In comparison, VarSome classified 67.3% (37/55) as PV/LPV, 30.9% (17/55) as VUS, and 1.8% (1/55) as LBV. For variants classified as BV/LBV by more than 50% of the ClinVar submitters, 81.8% and 72.7% were classified the same by VarSome and CanVIG-UK, respectively.

### 3.5. Comparative Analysis of Variants Updated to Consensus in 2024 ClinVar

Further analysis of the 450 conflicting variants in ClinVar on 1 May 2024 showed that 17 variants (3.8%) had reached a consensus (9 PV/LPV, 1 VUS, and 7 BV/LBV). The variant classifications of VarSome and CanVIG-UK were in agreement with the final ClinVar consensus for all but one variant each: one variant was classified as VUS by VarSome but as PV/LPV by ClinVar, and another classified as VUS by CanVIG-UK but as BV/LBV by ClinVar (Table 3). There was a consensus in 2024 that the BRCA1 variant c.5408G>C (p.Gly1803Ala) was an LPV, but VarSome still listed it as a VUS. The primary reason for this discrepancy is the absence of applied functional evidence in VarSome classification. Conversely, CanVIG-UK continues to interpret the variant c.5585A>T (p.His1862Leu), classified as BV/LBV by ClinVar in 2024 as a VUS. This conservative interpretation was due to the presence of two pathogenic supporting evidence criteria, despite functional studies indicating no impact (detailed data in Table S1).

## 4. Discussion

This study presents a comparative analysis of two tools, VarSome and CanVIG-UK, and focuses on the reinterpretation of *BRCA1* missense variants with conflicting classifications in the ClinVar database. The overall concordance rate between VarSome and CanVIG-UK was 58.9%, with similar PV/LPV classifications (11.3% and 11.1%, respectively). No variants classified as PV/LPV in VarSome were classified as BV/LBV in CanVIG-UK, or vice versa. VarSome classified more variants as BV/LBV (64.0%), whereas CanVIG-UK classified more variants as VUS (51.6%).

The differences in the results between the two tools may be due to their different approaches to variant interpretation. VarSome uses a generalized approach to access more genes. It provides a comprehensive platform for variant annotation and interpretation by integrating more than 140 data sources and can automatically classify variants based on the ACMG guidelines [8]. It classifies variants of multiple genes by integrating various data types and algorithms, making it suitable for large-scale analyses. Thus, VarSome can improve the consistency of variant interpretation across various genes; however, expert judgment remains important, especially when considering the specific characteristics of each gene and disease [20]. This suggests that an integrated approach that combines expert knowledge with automated tools is required.

In contrast, *BRCA1/BRCA2* CanVIG-UK offers a gene-specific approach tailored to *BRCA1* and *BRCA2* with detailed criteria emphasizing functional domains, functional study data, and population frequencies unique to *BRCA1*. CanVIG-UK prioritizes functional protein studies and provides detailed guidelines for assessing various types of evidence [12]. The CanVIG-UK framework provides specific references for functional evidence, which can be used as criteria for pathogenicity or benignity. This allows variants classified as VUS in VarSome, due to a lack of applied functional evidence, to potentially be reclassified as PV/LPV or BV/LBV. Through the application of such evidence, when at least one submitter reported a variant as PV/LPV, CanVIG-UK guidelines enabled most of these variants to be classified as PV/LPV (see Table 2). This reclassification was achieved by incorporating specific and diverse pathogenic criteria, including functional evidence codes, resulting in a classification more consistently aligned with pathogenicity. Conversely, 11 variants classified as PV or LPV in VarSome were assigned a BS3 functional evidence code in CanVIG-UK, indicating no significant impact on protein function, and thus remained classified as VUS in CanVIG-UK (Figure 2). This discrepancy raised questions about the pathogenic interpretation of variants that VarSome classified as pathogenic, highlighting the impact of functional evidence on variant classification consistency between platforms.

With additional analysis, among the 153 variants classified as VUS by CanVIG-UK but categorized differently by VarSome, 90% (138/153) were identified as BV/LBV by VarSome (Figure 2). The key differences arose from the computational tools used for BP4 (benign computational predictions), with VarSome using MetaRNN, whereas CanVIG-UK relied on REVEL, which often produced inconsistent results. Furthermore, VarSome applied a range of evidence weights—from supporting to strong—whereas CanVIG-UK consistently applied only supporting strength. This difference in evidence weighting led to a high frequency of BV/LBV classifications in VarSome. Additionally, VarSome applied the BP3 criterion (in-frame deletions/insertions in a repetitive region without a known function) to 57 missense variants, a practice CanVIG-UK did not apply for missense variants, and extended the BP1 criterion to 27 variants, unlike CanVIG-UK. CanVIG-UK also incorporated splicing effects and domain-specific considerations when applying BP1. These variations in evidence application, tool selection, and evidence weight strength led to the observed classification discrepancies between VarSome and CanVIG-UK.

VarSome uses gnomAD generic databases for population frequency data, whereas CanVIG-UK recommends the use of gnomAD, a noncancer female Popmax Filtering AF with 95% confidence for minor allele frequencies, applying this standard while disregarding PM2 evidence when benignity is strongly supported. This gene-specific approach, similar to that of the ClinGen ENIGMA Consortium [21], helps elucidate the complexity and clinical relevance of *BRCA1* and *BRCA2* variants.

The different interpretations of these two methods have significant clinical implications. For example, variants classified as VUS by VarSome and as LPV by CanVIG-UK may lead to different clinical decisions [22,23,24]. A VUS classification often results in a more cautious approach, treating the variant as a marker of uncertain significance. This leads clinicians to delay proactive interventions, such as preventive surgeries, until further evidence is available, potentially delaying the timely management of at-risk individuals. Conversely, an LPV classification suggests a probable pathogenic association, encouraging more assertive clinical actions. These include prophylactic surgeries, targeted screening protocols, and family cascade testing to identify at-risk relatives.

In this study, further analysis showed that some conflicting variants in ClinVar reached a consensus classification by May 2024, highlighting the importance of the regular re-evaluation of variant classifications in clinical practice. Systematically comparing conflicting classifications and integrating re-evaluated outcomes can support the development of optimized, consensus-driven conclusions. Moreover, by cross-referencing the classifications between the two platforms, clinicians can gain a more comprehensive understanding of each variant’s significance, facilitating more informed decision making. Adopting harmonized guidelines and fostering collaboration across classification platforms can minimize discrepancies, thereby enhancing patient care and ensuring more consistent clinical recommendations.

The main strength of this study is that it compares two commonly used tools for predicting the effect of conflicting *BRCA1* missense variants, which helps assess the performance of these methods. However, this study has several limitations. The use of automated classification by VarSome without manual intervention may result in some details being missed. In addition, VarSome and CanVIG-UK are regularly updated to reflect the latest information. A more comprehensive guideline was published by ClinGen when this study was designed; however, it was not included [21]. Therefore, the variant interpretations described here reflect the guidelines available at the time of analysis and may change with future updates. Future research should include more up-to-date guidelines, such as the updated ClinGen ENIGMA recommendations, to improve variant interpretation procedures and increase clinical accuracy [21]. This ensures that patients receive the most accurate and up-to-date information through the regular re-evaluation of variant classifications using the latest tools. Finally, this study focused only on *BRCA1* missense variants and did not include other types of variants, such as splicing variants or insertions/deletions, which could require the application of distinct evidence codes for interpretation. Including these variants in future studies could extend the applicability of our finding.

## 5. Conclusions

We evaluated the performance of VarSome and CanVIG-UK in reinterpreting *BRCA1* missense variants. This study shows that variant interpretations may differ depending on the method or tool used, highlighting the need for better and more accurate interpretation frameworks. In cases wherein consensus on variant classification is lacking, the adoption of a combined approach that incorporates both broad generalized methods and gene-specific guidelines may provide a more informed and accurate basis for clinical decision making.

## Figures and Tables

**Table 1:** Variant classifications by VarSome and CanVIG-UK for 450 conflicting missense variants in ClinVar.

Classification (=450)	VarSome %	(No.)	CanVIG-UK %	(No.)
PV/LPV	11.3	(51)	11.1	(50)
VUS	24.7	(111)	51.6	(232)
BV/LBV	64.0	(288)	37.3	(168)

Abbreviations: PV, pathogenic variant; LPV, likely pathogenic variant; LBV, likely benign variant; BV, benign variant; VUS, variant of uncertain significance. CanVIG-UK, *BRCA1/BRCA2*: CanVIG-UK gene-specific guidance.

**Table 2:** Proportion of VarSome and CanVIG-UK classifications in relation to ClinVar submitter results.

ClinVar, Submit as PV or LPV Per Variant	VarSome, PV or LPV % (No.)		CanVIG-UK, PV or LPV % (No.)	
>50% of submitters (*n* = 21)	90.5	(19)	95.2	(20)
≤50% of submitters (*n* = 34)	52.9	(18)	88.2	(30)
at least 1 submitter (*n* = 55)	67.3	(37)	90.9	(50)
**ClinVar, submit as BV or LBV per variant**	**VarSome, BV or LBV % (No.)**		**CanVIG-UK, BV or LBV % (No.)**	
>50% of submitters (*n* = 66)	81.8	(54)	72.7	(48)
≤50% of submitters (*n* = 329)	70.8	(233)	36.5	(120)
at least 1 submitter (*n* = 395)	72.7	(287)	42.5	(168)

Abbreviations: PV, pathogenic variant; LPV, likely pathogenic variant; LBV, likely benign variant; BV, benign variant; VUS, variant of uncertain significance. CanVIG-UK, *BRCA1/BRCA2*: CanVIG-UK gene-specific guidance.

**Table 3:** Comparison of 17 conflicting ClinVar missense variants that reached a consensus classification (assessed on May 2024) with VarSome and CanVIG-UK interpretations.

No.	Variant Description	ClinVar Data (Accessed December 2022)				Interpretation		ClinVar Update Data (Accessed May 2024)
		Total No. of Submissions	PV/LPV (%)	VUS (%)	LBV/BV (%)	VarSome	CanVIG-UK	
1	c.811G>A (p.Val271Met)	15	0.0	6.7	93.3	LBV	LBV	BV/LBV
2	c.2207A>C (p.Glu736Ala)	7	0.0	14.3	85.7	LBV	LBV	BV/LBV
3	c.2735A>G (p.Lys912Arg)	6	0.0	16.7	83.3	LBV	LBV	LBV
4	c.4766G>A (p.Arg1589His)	6	0.0	16.7	83.3	LBV	LBV	LBV
5	c.3818A>G (p.Gln1273Arg)	4	0.0	25.0	75.0	LBV	LBV	LBV
6	c.5585A>T (p.His1862Leu)	4	0.0	25.0	75.0	LBV	**VUS**	BV/LBV
7	c.2155A>G (p.Lys719Glu)	13	0.0	61.5	38.5	LBV	LBV	BV
8	c.441G>C (p.Leu147Phe)	7	14.3	85.7	0.0	VUS	VUS	VUS
9	c.5521A>C (p.Ser1841Arg)	5	40.0	60.0	0.0	LPV	LPV	LPV
10	c.5165C>A (p.Ser1722Tyr)	4	50.0	50.0	0.0	LPV	LPV	LPV
11	c.5254G>C (p.Ala1752Pro)	2	50.0	50.0	0.0	LPV	LPV	PV/LPV
12	c.5362G>T (p.Gly1788Cys)	2	50.0	50.0	0.0	PV	LPV	LPV
13	c.5090G>A (p.Cys1697Tyr)	3	66.7	33.3	0.0	PV	LPV	LPV
14	c.5258G>C (p.Arg1753Thr)	3	66.7	33.3	0.0	LP	LPV	PV/LPV
15	c.5143A>T (p.Ser1715Cys)	5	80.0	20.0	0.0	PV	PV	LPV
16	c.5408G>C (p.Gly1803Ala)	5	80.0	20.0	0.0	**VUS**	LPV	PV/LPV
17	c.5332G>A (p.Asp1778Asn)	6	83.3	16.7	0.0	PV	LPV	PV/LPV

Abbreviations: PV, pathogenic variant; LPV, likely pathogenic variant; LBV, likely benign variant; BV, benign variant; VUS, variant of uncertain significance. CanVIG-UK, *BRCA1/BRCA2*: CanVIG-UK gene-specific guidance.

## Data Availability

The datasets used in the current analysis are available from the corresponding author upon reasonable request.

## Supplementary Materials

The following supporting information can be downloaded at https://www.mdpi.com/article/10.3390/diagnostics14242821/s1: Figure S1: Distribution of 450 conflicting *BRCA1* missense variants in ClinVar (accessed 20 December 2022). Table S1: List of 450 conflicting ClinVar *BRCA1* missense variants analyzed using VarSome and CanVIG-UK. Table S2: Interpretation details on classification discrepancies of 15 *BRCA1* variants: PV or LPV by VarSome vs. VUS by CanVIG-UK. Table S3: Interpretation details on classification discrepancies of 14 *BRCA1* variants: VUS by VarSome vs. LPV by CanVIG-UK.
