# Supplementary material for: Reinterpretation of Conflicting ClinVar BRCA1 Missense Variants Using VarSome and CanVIG-UK Gene-Specific Guidance
Source: Diagnostics (Basel). 2024 Dec 14;14(24):2821. doi: 10.3390/diagnostics14242821 (PMC11675547; doi:10.3390/diagnostics14242821)
Supplement: Supplementary file 1 [file diagnostics-14-02821-s001.zip › diagnostics-3307629_Supplementary_Figure S1.pdf]

SUPPLEMENTARY MATERIALS

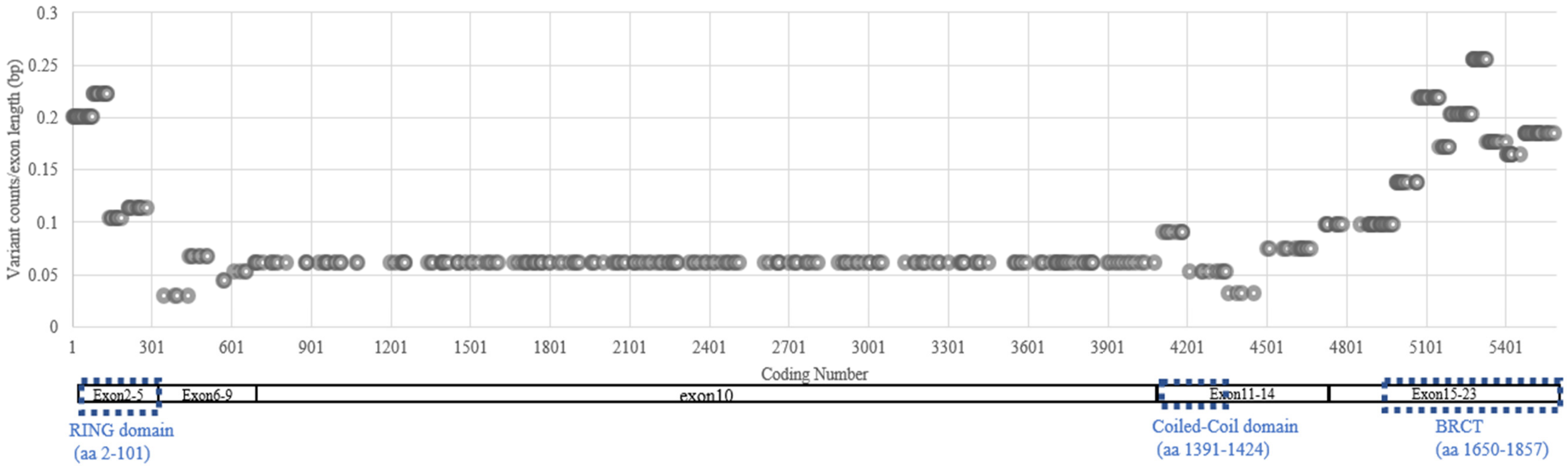

Figure S1. Distribution of 450 conflicting missense variants of the *BRCA1* gene in ClinVar (accessed December 20, 2022)
